# Urban foraging: Land management policy, perspectives, and potential

**DOI:** 10.1371/journal.pone.0230693

**Published:** 2020-04-07

**Authors:** Mallika Sardeshpande, Charlie Shackleton

**Affiliations:** Rhodes University, Grahamstown, South Africa; University of Bucharest, ROMANIA

## Abstract

Gathering of uncultivated food from green spaces, also known as foraging, is observed in urban areas across the world, but the literature focuses predominantly on the global north. Our study examines the existing urban land management structure and its approach to urban foraging in the eastern coastal region of South Africa. Through interviews with municipal officials in nine cities, we identified different stakeholders and their roles in urban green space management. We then used network analysis to represent interactions and influence of these stakeholders, and environmental worldviews to determine organisational and perceptual barriers to and enablers of foraging in urban green spaces. The policy on urban green space management, as well as land managers themselves are amenable to the concept of foraging in public spaces. Lack of knowledge on wild indigenous species and sustainable offtake, ambiguous, coarse, or lacking policy, and normative views of pristine nature may hinder foraging. We recommend pathways for policy and stakeholder partnerships to incorporate sustainable foraging in their biodiversity conservation and land stewardship strategies.

## 1. Introduction

Urban open space is a valuable resource globally, with over half of the world’s population residing in cities, and an annual urbanisation rate of 1% in developing and middle-income nations [1]. In the face of densification and development, urban green space is a critical yet contested component of the urban landscape [2, 3]. Urban green space constitutes predominantly undeveloped space within urban and peri-urban limits that supports multiple ecological and social processes [4]. It includes vegetation surrounding managed structures such as roofs, power lines, and verges [5, 6], managed formal spaces such as public parks, gardens and forests [7], unmanaged informal spaces such as vacant lots and edges [5], as well as urban forests [8]. It provides numerous ecosystem services such as macro- and micro-climate regulation and resilience [6, 8], biodiversity conservation and connectivity [9, 10], and cultural and recreational value [11–13].

A widely recognised but relatively understudied service of urban green infrastructure is the provisioning of natural resources such as medicinal herbs [14], wild foods [15], and fuel wood, for subsistence as well as cash income [16]. Foraging is the activity of collecting such resources from the natural environment [17], that are not cultivated or farmed commercially. Urban foraging in developed nations tends to be linked to cultural traditions, place-making, and improved quality of living [15,18,19] and in some cases, also with subversive ideological movements [20–22], but motivations for the same in developing nations are underreported. Foraging in developing cities may contribute significantly to urban poverty alleviation [23], contributing on average 20% of the household income among the urban poor [24]. Implicitly, the prevalence of foraging is contingent upon household access to green spaces [25, 26]. Citizen access to urban green spaces may be constrained by uninformed planning [7, 27], socioeconomic bias [28], historical legacy [29], and ambiguous policy [30].

Urban foraging holds potential as a citizen stewardship strategy, by supporting devolved governance, informal green space co-management, and urban biodiversity conservation [31–34]. However, the links between foraging and urban green space management remain unclear, as public engagement in urban green space management may be arbitrary, inconsistent, or unorganised [35, 36], and extractive use of formal public spaces is often deemed unlawful by policy [37, 38]. Within the current decade, cities in developed nations have begun to formulate policies with the specific aim of promoting public edible landscapes [39]. The current literature on urban green spaces and foraging is predominantly from the global north [5, 3, 40], and is lacking representation from Africa, where foraging is common and urban centres are fast growing [2, 17, 41].

Our study fills the gap in knowledge on the policy response to urban foraging in formal and informal urban green spaces in South Africa. Our primary research questions are: (i) what are the different types of urban green spaces and the institutions and policies governing them? (ii) do these policies or institutions address the phenomenon of foraging; if yes, how, and if no, how would they? (iii) can foraging as an urban green space use also contribute to landscape stewardship, why, and how? (iv) what are the potential enablers and barriers to foraging in urban green spaces? Through semi-structured interviews with urban land managers, we map the existing policies, practices, and partnerships in urban green space management, and ascertain if urban foraging is in conflict or concord with these. We use network analysis to identify the key stakeholders in urban green space management, and the relationships between them. We also use an environmental worldviews framework to explore the links between urban green space managers’ normative views about nature and their level of agreement with foraging.

Network analysis is a technique from information science [42] that has been used in conservation to identify key stakeholders [43–45] and the flow of information and trust between them [46], land use change drivers [47], habitat trees [48], and conservation planning [49] and development [50]. We use it to identify important urban land users and managers, and through them, the potential points of entry for planting and uptake of wild edible fruit species and forager-manager partnerships. Environmental worldviews are known to influence people’s behaviour towards and use of natural resources [51, 52], and may also influence decisions by policy-makers and land managers [53, 54]. We use an adaptation of the Future of Conservation framework [55] to evaluate urban green space managers’ perspectives on nature conservation, and assess if these influence their response (as barriers or enablers) to urban foraging.

### 1.1 Study area

The study area, the Indian Ocean Coastal Belt (IOCB), is host to rich biodiversity [56] and dense human population, is undergoing rapid land cover change [57], and urban foraging is highly prevalent in this region (Sardeshpande and Shackleton unpublished data). We focus on the planting and foraging of wild edible fruit species, because they are (i) widely foraged globally [58], within South Africa [59], and in the study area (Sardeshpande and Shackleton unpublished data), (ii) commonly found in the wild as well as in human-dominated landscapes including agroforestry systems, home gardens, and urban green spaces [60], (iii) significant contributors to nutrition and income across the socioeconomic and rural-urban gradient [23], (iv) often resilient to climatic and harvesting pressures [61], and (v) potentially important to urban biodiversity, particularly frugivores and pollinators [62]. South Africa is among the most unequal economies in the world ([63], p. 21), with a quarter of its population living below the food poverty line ([63], p. 14). Its history of racial segregation has resulted in uneven distribution of infrastructure, including urban green spaces [64]. This juxtaposition of food insecurity, unequal access to urban green spaces, and prevalence of foraging makes a compelling case for further investigation of the enablers of and barriers to improved food security and green space provisioning and management through urban foraging.

## 2. Methods

### 2.1. Site selection

The IOCB houses the metropolitan municipality of Durban, along with 10 other urban local municipalities in five districts [65]. The populations of these municipalities range from about 120,000 (KwaMbonambi) to 3.4 million (Durban) people [66], with a mean population of 492,720 people. Community services, parks, and environment departments in all 11 municipalities were contacted by email. Where no response was received, the municipal offices were visited for an appointment to speak with relevant officials. Full length interviews were conducted in eight of the 11 municipalities (Table 1), while officials were not available for interviews in the other three.

**Table 1:** Details of the key informants interviewed.

Town/City	Parks Manager	Environmental Manager	Community Services	Total
Kwambonambi	NA	NA	1	1
Richards Bay & Empangeni	1	1	1	3
Eshowe	NA	NA	1	1
Stanger & Ballito	1	1	0	2
Durban	1	2	1	4
Scottburgh & Park Rynie	1	1	0	2
Port Shepstone	1	0	0	1
Port St Johns	NA	NA	1	1

### 2.2. Semi-structured interviews

Key informants were initially identified within each municipality from municipal websites and contacted with a request for an interview. In municipalities with a designated parks department or environment department, parks managers and environmental managers were interviewed. In municipalities without designated parks or environment departments, officials in charge of tree planting and open space or environmental management within the community services department were interviewed. In the metropolitan municipality of Durban, we also interviewed an additional parks horticulturist and agroecology horticulturist as their role in managing open spaces was seen as pivotal by their colleagues in the parks and environment departments. A total of 15 key informant municipal officials were interviewed (Table 1). We consider this a representative sample size based on the specificity of our research aims and questions, the strength of dialogue and density of information, and the case-based analyses of the qualitative data [67], yielding sufficient information power. This study was approved by the Rhodes University ethics committee (Application No. ES17/46). As per the ethics approval, oral consent was obtained to record the interview, and the data were anonymized.

Interviews with key informants were guided by the set of research questions (S1 Appendix) about the scope and extent of formal and informal public open spaces under their administration; their mandate, management practices, and policies; and their opinion on foraging in public open spaces. Of the 15 key informants, 13 were asked to answer Likert scale questions about their environmental world views (the remaining two key informants deemed themselves unsuitable for comment as they managed broader mandates of community services). Interviews were conducted on an individual basis to avoid groupthink and collective answers. Interview length varied from 14 to 65 minutes, with a mean duration of 35 minutes. All interviews were recorded as audio files, and completely transcribed in MS Word. Respondents were labelled as CS (community services), EM (environmental manager), and PM (parks manager) for reporting purposes.

### 2.3. Data analysis

#### 2.3.1. Qualitative analysis

Transcripts were coded for emergent themes manually in MS Excel. Information from interviews was complemented and triangulated with that from the Integrated Development Plans (IDPs) and Spatial Development Frameworks (SDFs) of respective municipalities as available. All 11 municipalities had some documents available online, as a result of which the three municipalities unrepresented by key informants were included in some comparisons (Table 3). Specifically, searches were run for figures (number, budget, area, etc.) and policies about parks, conservation areas, and open spaces. These documents did not contain any information on management protocols.

#### 2.3.2. Network analysis

The links between stakeholders were ascertained from the interviewee responses. Although no directed questions about stakeholder links were included in the initial interview guide, this information invariably and spontaneously surfaced in the interviews. Based on this data, a network was developed to identify the key actors and their connections to urban open spaces. Information from the interviews was used to map networks of stakeholders in urban open space management. Each group of actors involved in urban open space management was defined as a vertex or node, and the flow of management actions between these actors was considered an edge. The networks were directed and weighted (e.g. [68]), and weights were based on the nature of transfers between stakeholders. Four types of management actions were identified, each regarded independent of the others, and assigned a weight (Table 2). The identification, independence, and weight of actions were a posteriori assumptions made by the authors to conceptually represent the networks. As the aim of the analysis was to determine the degree of influence of actors on land management, direct management actions were assigned maximum weight, and devolved management rights, minimum weight.

**Table 2:** Types of management actions and weights assigned to them.

Management actions	Weight
Approval or permission to use land (e.g. EIA approval)	1
Sharing of advice and expertise on land use management (e.g. landscaping advice)	2
Provision of management services (e.g. invasive alien control)	3
Sponsorship of plants, equipment, funding (e.g. offsets)	4

Networks were created and graphs plotted in R using the ‘igraph’ package. Degree centrality and authority and hub scores were calculated in R. Degree centrality is the absolute number of edges that a given vertex or node has with other all other vertices or nodes within the network. An authority is a vertex or node with the most incoming edges, and a hub is a vertex or node with the most outgoing edges. Thus, degree centrality identifies the actors with the most connections, and authority and hub scores identify the actors that receive and provide maximum input in urban open space management, respectively.

#### 2.3.3. Environmental worldviews

Holmes et al. [69] developed a set of 28 Likert scale questions, based on the New Ecological Paradigm [70], the Inclusion of Self in Nature [71], the Two Main Ecological Values [72], and other frameworks, to conceptualise environmental worldviews of conservation workers. Sandbrook et al. [55] classify these worldviews into four quadrants, each representing a different but not mutually exclusive approach to conservation:

- Critical Social Science (e.g. [73]), leaning towards ethical anthropocentricism
- Traditional Conservation (e.g. [74]), based on preservationism
- New Conservation (e.g. [75]), tending towards utilitarianism
- Market Biocentrism (e.g. [76]), advocating market-ecosystem segregation

In our adaptation of the Future of Conservation framework, the essence of 38 functional and normative assertions is distilled into seven normative statements, and respondents are asked to choose whether they agree, disagree, or feel neutrally about these statements. This compression reduces repetition (and potential respondent fatigue), breaks down complex conservation concepts into simple layperson terms, and avoids radical or abstract stances that respondents may not understand or may respond aversely to. The reduction in questions renders the scale susceptible to low internal validity, and increases the coarseness of response translation into worldview categories. However, we do not classify respondents into categories, but only identify their beliefs, and test for statistical differences between their worldviews and their opinion of foraging.

Environmental worldviews of interviewees were compared to their stance on urban foraging using a Chi-squared test with the null hypothesis: The interviewee’s stance on foraging is independent of their environmental worldviews. We acknowledge that the assumption of a minimum expected frequency of 5 is violated by our dataset. Chi-squared tests were performed in R 3.6.0 [77].

## 3. Results

### 3.1. The nature and number of open spaces

Of the 11 municipalities, four had formal open spaces, and four had conservation areas that are demarcated as such in town planning schemes (Table 3). Formal open spaces (parks and gardens) are characterised by landscaping features and amenities such as seating to facilitate public access and recreation. These are distinguished from informal open spaces, which lack improvement or demarcation, but are often used by the public for recreational purposes, and are viewed by municipalities as areas for future development and conversion to formal parks (CS2, PM3, CS3, PM5, EM3, CS4). Informal open spaces were identified and defined in the town planning schemes of eight out of the 11 municipalities. Some open spaces are demarcated for conservation purposes due to their strategic location (EM3), biodiversity (EM4), and ecosystem services (EM5). Such conservation areas are jointly managed by municipalities and citizens, and are mostly open for public use, subject to environmental considerations and at times user fees. Seven of the 11 municipalities host formally protected areas, although only Durban has protected areas administered by the municipality (the rest fall under provincial administration).

**Table 3:** The number and nature of open spaces across urban municipalities in the study area.

Town/City Name	Formal Open Spaces	Informal Open Spaces	Protected Areas	Department in charge
Mtubatuba^1^	0	4	2 [Provincial]	Community Services
KwaMbonambi^2^	0	3	0	Community Services
Richards Bay & Empangeni^3^	2	4 [Conservation]	0	Parks, Environment
Eshowe^4^	0	5	1 [Provincial]	Community Services
Stanger & Ballito^5^	0	5 [Conservation]	2 [Provincial]	Parks, Environment
Mandini	0	Undefined	2 [Provincial]	Community Services
Ndwedwe^6^	0	Undefined	0	Community Services
Durban^7^	12	10	7	Parks, Environment
Scottburgh & Park Rynie^8^	2	11 [Conservation]	0	Parks, Environment
Port Shepstone^9^	3	17	7 [Provincial]	Parks, Environment
Port St Johns^10^	1	Undefined	1 [Provincial]	Community Services

1 Mtubatuba SDF [78]

2 Umfolozi Municipality [79]

3 Umhlathuze SDF [80]

4 Umlalazi Municipality [81]

5 Quayle and Pringle [82]

6 Sishi, N (pers. comm.)

7 Govender [83]

8 Umdoni IDP [84]

9 Ray Nkonyeni IDP [85]

10 Port St Johns IDP [86].

Out of the 11 municipalities, five had dedicated parks and environment departments to manage their open spaces, while the rest assigned this role to their respective community services departments. In Durban, the agroecology division played a significant role in management of all three types of open spaces (PM1, EM1, CS1). Parks departments in the different municipalities had between 100 and 300 staff members, and environment departments had between one and 25 staff members. Community services departments had between one and three staff members whose role related to open space management in the form of tree planting, waste management, or beach management. The agroecology division in Durban had seven staff members. The community services and agroecology departments engaged general workers from the government’s Extended Public Works Programme (EPWP) when required, such as for planting and clean-up campaigns (PM4, CS1).

### 3.2. Open space management: Practices, policies, and planting

The suite of management practices used in open spaces ranged from plant trimming and biomass removal to restoration and carbon and biodiversity offset greening (Table 4). Parks and environment departments often work closely with the waste management section within the municipality (PM4, CS5, EM3, EM4). While plant trimming, biomass removal, and landscaping are mostly undertaken by parks departments and their community service counterparts, planting is often a joint undertaking by the parks and environment departments. Parks and community services departments are usually trained in mechanical invasive alien control methods such as trimming (PM2, PM4), but often enlist specialist teams from the environment department or the district administration for chemical control or intensive removal (CS3, PM5). In areas where sensitive ecosystems are faced with high development pressures, the environment departments engage with developers and town planners to protect and restore open spaces. In Durban, the Botanic Gardens and the agroecology division promote permaculture practices.

**Table 4:** Management practices and departments undertaking them.

City Name	Trimming & biomass removal	Planting	Invasive alien control	Awareness & education	EIA, planning, protection	Compliance, offsets, restoration
Mtubatuba	Community Services	Community Services	NA	Community Services	NA	NA
KwaMbonambi	Community Services	Community Services	Community Services	Community Services	NA	NA
Richards Bay & Empangeni	Parks	Parks + Environment	Parks + Environment	Community Services	Environment	Environment
Eshowe	Community Services	Community Services	NA	NA	NA	NA
Stanger & Ballito	Parks	Parks + Environment	Parks + Environment	Environment	Environment	Environment
Durban	Parks	Parks	Environment	Parks + Environment	Environment	Environment
Scottburgh & Park Rynie	Parks	Parks + Environment	Parks + Environment	NA	Environment	Environment
Port Shepstone	Parks	Parks + Environment	Environment	NA	Environment	NA
Port St Johns	Community Services	Community Services	Community Services	NA	NA	NA

Conservation areas are characterised by a focus on the conservation of ecosystem integrity and services, thereby subject to well-defined rules on the kinds of public uses that can and cannot be undertaken within them. Only the formal parks and gardens in Durban have clearly defined rules and policies for the kind of activities and uses permitted inside them. These tie in with the larger framework of the Durban Metropolitan Open Space System (DMOSS) that governs land use throughout the municipality in formal, informal, and protected open spaces (EM1, EM2, CS1, PM1). Other municipalities with similar policies around informal open space use are Richards Bay and Empangeni’s Environmental Services Management Plan (ESMP) (EM5), Stanger and Ballito’s Biodiversity and Open Spaces Map (BOSMap) (EM3), and Scottburgh and Park Rynie’s Tree Policy (EM4). These policies help the municipalities to prioritise land for conservation, development, and a spectrum of intermediate land uses. In Durban, the Botanic Gardens and Silverglen Nature Reserve are two examples of specific use policies. The Durban Botanic Gardens allows visitors to extract biological material, usually for research purposes, only with the recorded authorisation of the administration (PM1). The nursery at Silverglen Nature Reserve offers its visitors and traditional healers the opportunity to extract biological material such as tree bark, generally for personal or professional use (PM2, Oxland, J. pers. comm.).

While the DMOSS does not explicitly address the harvest of wild fruits or animals as a source of food, it does refer to the removal of bark from trees for medicinal use. Such use is generally permissible so long as it is done without inflicting significant damage to the plant (EM2, EM1). Similarly, the ESMP does not mention the use of wild fruits or food, but does acknowledge the use of natural resources such as reeds from urban open spaces, and incorporates such use as an ecosystem service that contributes to the biodiversity economy, thereby advocating sustainable use (EM5). The BOSMap considers more widely the sensitivity of ecosystems and their services, not delving into specific small-scale uses such as wild food foraging (EM3). The Tree Policy similarly advises on priority ecosystems and species, not focusing on the details of natural resource extraction (EM4). The existing policies on open space use are either ambivalent or encouraging of natural resource extraction provided it is sustainably done.

Planting of trees is actively undertaken by most municipalities during Arbour Week in September. Municipalities with parks and environment departments have internal budgets allocated to planting, although officials admitted it was difficult to provide a figure for how much of the budget was spent on planting or how many trees were planted (Table 5). In smaller municipalities, planting was undertaken incidentally, such as when a new housing development required greening (CS3), or when private entities initiated offset greening (EM4). Most municipalities supplement their internal planting budget with sponsorship from the Department of Agriculture, Forestry and Fisheries (DAFF) and the Department of Economic Development, Tourism, and Environmental Affairs (EDTEA). Where restoration or offset plantation is undertaken, the developers and occasionally, NGOs like Wildland Conservation Trust (WCT), sponsor the purchase of plants. Planting is generally done in low income housing yards, schools, and newly developed sidewalks and amenities. In Eshowe, trees planted by the municipality in public open spaces were reportedly stolen within a week (CS2), so the officials decided to plant in household yards rather than openly accessible spaces. In some cases, verges and sidewalks in low income housing neighbourhoods may be too narrow and already occupied by water and electricity services (PM2), prompting planting inside yards rather than in open spaces. The sponsors and location of the planting often influence the choice of species planted, although Arbour Week plantings commonly consist of one (horticultural, domesticated) fruit tree and one indigenous shade tree per household.

**Table 5:** Factors influencing planting in different municipalities in the study area.

Town/City Name	List of prescribed planting species	Annual planting frequency	Budget sources	Target location	Species selection criteria
Mtubatuba	None	Unknown	Unknown	Unknown	Unknown
KwaMbonambi	None	Incidental	Infrastructure, EDTEA, WCT	Small town rehabilitation, sidewalks	Guidance & sponsorship from EDTEA, WCT
Richards Bay & Empangeni	None	Once (Arbour Week)	R600,000* Internal, DAFF, EDTEA	Low income housing yards, open spaces, restoration	Indigenous, drought resistant, shade; fruit, vegetables
Eshowe	None	Once (Arbour Week)	R30,000 Finance, DAFF, EPIP	Low income housing yards, schools	Fruit, shade, indigenous, succulents; guidance & sponsorship from DAFF, EPIP
Stanger & Ballito	Yes	Monthly (Annual total 400 plants)	R200,000 Internal, DAFF, EDTEA	Low income housing yards, schools, open spaces, restoration	What suits the area; indigenous, fruit; non-aggressive growth and fruiting; medium maturity size; non-messy trees; history
Durban	Yes	Unknown	R416,00,000*	Low income housing amenities, open spaces, schools, restoration	Sponsorship from WCT +
Scottburgh & Park Rynie	Yes	Incidental	Internal, DAFF, EDTEA	Low income housing amenities, restoration	Guidance & sponsorship from DAFF, EDTEA
Port Shepstone	Yes	Incidental	R600,000* Internal	Low income housing amenities, open spaces, schools	Indigenous, ornamentals, guidance & sponsorship from DAFF, EDTEA
Port St Johns	None	Unknown	R20,000 Internal	Unknown	Unknown

* these are budgets for Parks development and maintenance, of which plantings are one part

DAFF: Department of Agriculture, Forestry and Fisheries, EDTEA: Department of Economic Development, Tourism, and Environmental Affairs, WCT: Wildlands Conservation Trust

The species lists from the parks departments contained a number of medicinal species, as well as *Trichelia dredgeana*, a commonly occurring species whose arils are often consumed as a delicacy after soaking in water. However, each species list had only two wild edible fruit species, namely *Harpephyllum caffrum* (appeared in two lists), *Diospyros whyteana* (appeared in one list), and *Ficus sur* (appeared in one list). The agroecology species list did not contain any wild edible fruit species. There was also a recurring perception among interviewees that edible fruit species were either not wild or not indigenous, and that commonly occurring plant species in open spaces were either invasive alien guavas or not fruit bearing.

*‘Personally I wouldn’t [encourage people to forage] because it is very difficult to differentiate between indigenous and alien fruits…Fruits from alien plants are poisonous*’

- CS3

*‘Guava is alien*, *mango and pear are exotic*. *The waterberry* (Syzygium spp)*… I haven’t seen fruits on it*. *You get a black-purple fruit* (Flacourtia indica)*… but that tree is not indigenous*.*’*

- EM4

*‘We’ve been getting a lot of comments from new developers… about can we plant fruit trees along the sidewalks in our developments*. *And our main issue with that is that so many of the fruit trees are not indigenous*. *Meaning that they use a lot more water… and we’re going to be water scarce in the future so there’s serious issues with that*.*’*

- EM1

However, on further discussion, most interviewees recollected examples of indigenous wild plant species that bear edible fruit. There is scope for the inclusion of more wild edible fruit species in plantings, and municipal officials are open to the prospect of such. Lack of information coupled with budget restrictions may be the only obstacle in the uptake of wild edible fruit species planting.

### 3.3. Open space management: Stakeholder engagement and partnerships

Community services departments often undertake awareness and cleanliness campaigns to educate and engage citizens in waste management (CS5, CS3). Parks and environment departments partner with citizen bodies such as church and neighbourhood groups, conservancies, and urban improvement precincts as well as individual land owners to facilitate joint land management. Citizen groups may report on service delivery and illegal activities within their area, or organise as committees, non-profit organisations, or body corporates that facilitate the maintenance of public spaces, parks, or nature reserves through fundraising and vigilance with assistance from the municipality. In some cases, citizens and municipalities co-manage some public or private open spaces with the aim of conserving the biodiversity therein, although such areas are not formally developed or protected (see Table 3). Interviewees mentioned examples of 10 such partnerships in three municipalities, namely Durban (4), Scottburgh & Park Rynie (2), and Stanger and Ballito (4). Active public involvement in open space management exists in some municipalities, but is most established and evolved in Durban.

A branch of Durban’s environment department is dedicated to developing biodiversity stewardship partnerships with citizens. These partnerships offer land owners a suite of incentives in exchange for sustainable management of their land. These range from land zoning and environmental impact assessments for development, invasive alien control and vegetation burning in open spaces, to maintenance, management, marketing, and tax breaks in exchange for conservation servitudes. Through these partnerships and restoration programmes, the department has also developed citizen capacity in invasive alien control and management burning in some areas. The agroecology division in Durban focuses solely on citizen partnerships and capacity building. Interested citizens or groups approach the division seeking assistance in setting up their small-scale food production systems. The division helps them develop a co-operative, constitution, business plan, a memorandum of understanding with land owners, and linkages with fresh produce markets. They impart training in bio-intensive permaculture farming, and provide support in the form of seeds, saplings, and machinery. The Durban Botanic Gardens also hosts thematic programmes to educate the public about biodiversity and botanical heritage.

Environment departments in four municipalities undertake restoration and offset greening in partnership with developers. ESKOM, King Shaka International Airport, Tongaat Hulett Developers, TransNet, property developers, and sand mining companies were some of the examples cited. Strategic Environmental Assessments and Environmental Impact Assessments mandatory for development authorisation often stipulate compensatory greening, and municipalities team up with the DAFF, EDTEA, or NGOs to advise on the nature of restoration. Often such offset and restoration projects generate local biodiversity-based livelihoods such as *Papyrus* reed enterprise (EM5), and nursery and gardening enterprise (EM1). All interviewees involved in the execution of restoration projects agreed that including wild edible fruit species in such greening is feasible and holds potential for foraged fruit based livelihoods.

*‘We have mining houses… and I think urban foraging is one of those elements they’re gonna have to consider… [in] their rehabilitation efforts*.*’*

- EM5

### 3.4. Network analysis

**Fig 1:**
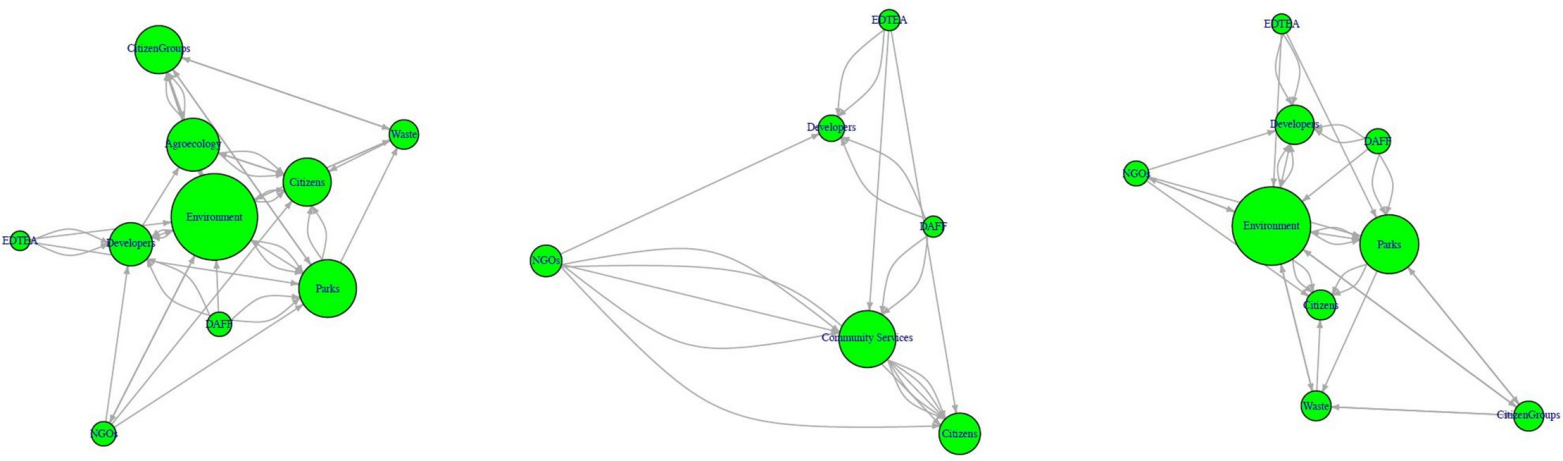
Network diagrams of stakeholders in open space management in: (a) metropolitan, (b) medium, (c) small urban municipalities. Arrows represent directed, weighted actions, and circle size represents degree centrality.

Up to 10 different stakeholder groups were identified (Table 6), although the number varied depending upon the size of the municipality. For example, whereas the municipality engaged with individual land-owner citizens as well as groups of citizens such as neighbourhood committees in Durban, medium-sized municipalities interacted with citizen groups, but seldom with individual citizens, and such citizen groups were not mentioned by interviewees in smaller municipalities. Therefore, three networks were constructed, one each for a metropolitan municipality (Durban), medium-sized urban municipalities (Stanger and Ballito, Port Shepstone, Richards Bay and Empangeni, Scottburgh and Park Rynie), and small urban municipalities (KwaMbonambi, Mtubatuba, Port St Johns, Eshowe). The networks were graphically plotted (Fig 1) to represent degree centrality (Table 6) of stakeholders in urban open space management. Each arrow between stakeholders represents a single, directed input action towards open space management (Table 2), and the size of the stakeholder circle represents the influence of the stakeholder in open space management.

**Table 6:** Degree centrality, and authority and hub scores of different stakeholders in different sized urban municipalities.

Stakeholder categories	Vertices or Nodes	Metropolis			Medium-sized towns			Small towns		
		Deg	Auth	Hub	Deg	Auth	Hub	Deg	Auth	Hub
Non-administrative	Citizens	10	1	0	6	0.55	0	8	1	0
	Citizen Groups	10	0.82	0.25	6	0.32	0.46	-	-	-
Local administration	Environment	18	0.52	0.73	16	0.64	1	11	0.84	0.66
	Parks	12	0.61	0.54	12	1	0.47			
	Waste	6	0.27	0.29	6	0.36	0.27			
	Agroecology	11	0.07	1	-	-	-	-	-	-
Provincial administration	DAFF	5	0	0.35	5	0	0.70	4	0	0.34
	EDTEA	4	0	0.31	4	0	0.62	4	0	0.48
Private sector	Developers	9	0.30	0.12	8	0.50	0.20	5	0.30	0
	NGOs	5	0.09	0.43	5	0.12	0.58	6	0	1

Deg: Degree centrality (total number of incoming and outgoing connections of stakeholder)

Auth: Authority score (rank of stakeholder relative to stakeholder with most outgoing connections)

Hub: Hub score (rank of stakeholder relative to stakeholder with most incoming connections)

While the environment and parks departments were central to open space management in the metropolitan and medium-sized municipalities, the community services department, that handled both these functions (albeit on much smaller scales) and waste management was central in the small municipalities (Table 6). The agroecology department in the metropolitan municipality also had a high degree centrality. Citizens were more central than developers and NGOs in the metropolitan and small municipalities, but developers were more central than citizens, who in turn were more central than NGOs in medium-sized municipalities. The DAFF and EDTEA were the least central entities in all three networks, as they only provide inputs to the local administration and private sector, but do not rely on them for any transfers.

Citizens and citizen groups were the authorities in the metropolitan municipality, receiving approvals, advice, management services, and sponsored plants from most other stakeholders (Table 6). The agroecology and environment departments in the metropolis were hubs that provided approvals, advice, services, and resources to citizens, the parks department, developers, and NGOs. In medium-sized municipalities, the parks departments were the authorities and the environment departments were the hubs, while citizens were the authorities and NGOs the hubs in smaller municipalities. The DAFF and EDTEA were hubs in medium-sized municipalities, often advising private sector stakeholders on restoration projects and sponsoring plants for parks and citizens.

### 3.5. Open space management: Challenges

**Fig 2:**
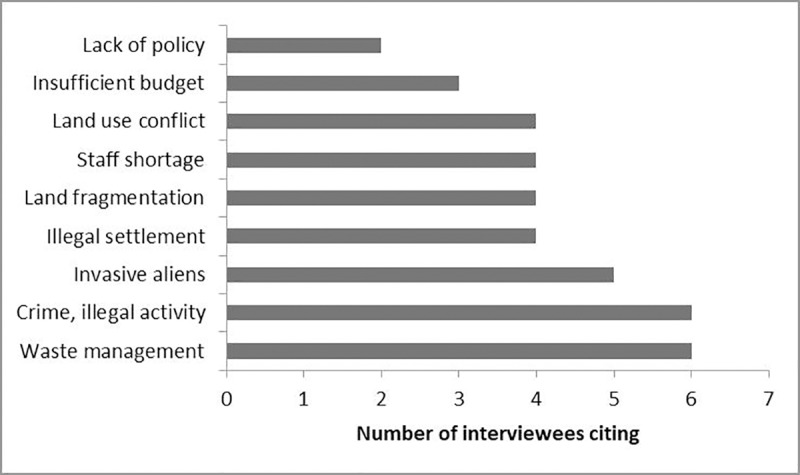
Challenges faced by land managers across municipalities (n = 15).

Illegal dumping was the single largest challenge in open space management cited by interviewees (7). Criminal and illegal activities such as drug use, sand mining, theft, and vandalism were also cited (7) as challenges, followed by invasive alien control (5). Illegal settlement by new urban immigrants, land fragmentation by agriculture, development, and land claims, land use conflicts between conservation and other development and recreational uses, and a shortage of staff were equally cited (4) challenges. Restricted budgets and lack of policy also figured on the list (Fig 2).

*‘Some say people are dumping because they are not aware*. *But I ask*, *if they are not aware*, *why don’t they dump right in front of the road*? *Why do they dump at night or during weekends*? *They know what they’re doing is wrong*. *It’s not a matter of awareness*, *it’s a matter of people being lazy*. *There’s no sense of responsibility*. *And contradictory to popular belief*, *all races are dumping*. *You catch them dumping*, *you’re like I thought you knew better*! *Just because people with money can afford [to live in upmarket suburbs] doesn’t mean they have that sense of responsibility*.*’*

- CS5

*‘Close to 2030 we will make sure that all our suburbs have parks in order to eliminate open spaces*. *Because people go to these spaces to [illegally] dump [waste]*, *the kids are busy with drugs*. *So once we develop those parks*, *it will assist us to reduce illegal dumping and [provide] recreation [opportunities] so [children] don’t pay attention to unnecessary things*.*’*

- PM5

*‘We don’t have big companies which contribute in terms of paying rate*. *We rely only on government budget which comes in the form of equitable share*. *So in budget we are restricted… and unfortunately due to limited budget*, *we are failing to fulfil our vision objective*.*’*

- CS2

*‘I don’t know why the municipality is keeping so much property*. *Because to maintain those [open spaces] is costly and time consuming*. *If you’re looking at certain areas within the municipality*, *there’s no place to plant a tree*. *And it gives us more work because the trees are falling*, *we’ve got to trim them*.*’*

- PM4

Besides these oft-cited challenges, some interviewees also highlighted issues stemming from the apartheid legacy. At the turn of the 20^th^ century, the Land Acts of South Africa dispossessed native peoples of their land, and displaced them into segregated, settlements with very poor living conditions and infrastructure [87]. With the abolition of apartheid in 1994, land was opened up to the economically underprivileged natives, in the form of government-sponsored low-cost housing in urban areas [88], and land in trusts governed by traditional chiefs in rural and peri-urban areas [89]. Open spaces in low-cost housing schemes have historically been under-provisioned [16,88, 90], and recent attempts at greening have in some cases been constrained by limited open space (PM2), or used exotic species (EM4). In the wider context of present-day open space management, traditional land claims and trusts may at times challenge or dispute land zoning for biodiversity conservation or public recreation (EM2, EM5). Particularly in Durban, where 44% of the land falls under the Ingonyama Trust, securing land for conservation is difficult due to differing priorities of the local municipality and the trust board (EM2).

*‘[The] spatial representation of nature reserves in Durban* … *it’s a very centralised representation*. *[A]ll the group areas people were shifted on to the periphery of the city where there were no opportunities*. *And then you get a new post-apartheid city where no one’s taking nature experiences to people*. *They’re just still at the central model* … *So when we try to work on the communal land*, *try to make inroads with the chiefs there*, *coming in and saying well we want a model where we want you to secure a portion of the land exclusively for conservation*, *and we don’t want people to use it*, *that model will never work in that context*. *We just wouldn’t get buy-in*. *So you have to have a different approach… an approach where it’s resource use*. *But in a way where hopefully those resources are being utilised in a manner that actually that’s sustainable*. *And people can see some benefits flowing from them*.*’*

- EM2

Lastly, some of the interviewees mentioned that it can be challenging to acquire, block, or retain suitable land for conservation, food production, and recreation in the face of other high-yielding economic uses like commercial or residential infrastructure (also see Environmental Worldviews).

*‘[O]ur biggest challenge here is land*. *Because more people want to [build] buildings now*. *Property developers [are] investing and pushing for houses*. *Plus they’re getting more monies*. *[For the] same land here*, *if they can pick up property for half a million or a million rand*, *why [would they] waste time [allowing people] to do small [permaculture] agriculture [on their land]*?*’*

- CS1

The municipalities face common challenges of waste, crime, and invasive alien species management that may be aggravated by the lack of financial or human resources and adequate policy responses. In addition, they acknowledge the need for improved planting in low-cost housing areas, and more inclusive use-based conservation models in traditional areas. Foraging has the potential to increase public use of open spaces, strengthening management capacity through citizen partnerships, and contributing to biodiversity enrichment in low-cost housing areas and conservation in traditional areas.

### 3.6. Foraging

Of the 15 interviewees, 12 reported to having observed some instance or evidence of foraging within their municipality, and 10 of these instances were of wild edible fruit. Medicinal bark removal (4), *Papyrus* reed harvest (1), and mushroom picking (1) were the non-fruit foraging instances reported. *Carissa* (4), *Eugenia* (1), *Passiflora* (2), *Psidium* (2), and *Syzygium* (2) were the wild species interviewees reported having seen foraged. Instances of informal vendors of these species were reported by four interviewees.

Twelve of the 15 interviewees were in favour of foraging, and three were concerned about its implications, namely foraging threatening vulnerable species (2), encouraging propagation of invasive alien species (2), and being a health hazard for the uninformed (2), which in turn would be a liability for the municipality or management.

*‘I don’t know if I would encourage people to [forage] because you see I will say I’m encouraging people to take those fruits… and then the whole species [is] gone*. *So I think those species somehow need some kind of protection… if they are available in public spaces… like if you do require to take some fruit… there must be some sort of permission from the municipality… so that it is not free for all*.*’*

- PM3

In protected and formal open spaces, foraging may be restricted to certain areas or species, and this information is usually provided on signboards or at the administrative offices of these spaces. For example, although foraging in the Durban Botanic Gardens is legal, the rules require that anybody extracting any plant material from the Gardens seeks permission from the management. In informal open spaces, this is generally not the case, unless the space is private.

*‘So to my mind*, *people coming and removing fruit from those [common] trees [in nature reserves]*, *they do it in any case*, *informally*. *Would you want to formalise it*? *I don’t know if it’s necessary*, *but there’s no one stopping them and I don’t think there’s a problem with them doing that*.*’*

- EM2

*‘If you’re taking from mother nature*, *it’s a good thing*. *My problem is against stealing [laughs]*. *[Foraging] is not stealing*, *because it’s an open space*. *Although it belongs to the municipality*, *it’s an open space*. *[People] coming to your property*, *jump[ing] over your fence*, *that is stealing*.*’*

- PM4

Three of the 12 interviewees in favour of foraging said it would augment food supply and security. In terms of managing open spaces, those in favour of foraging said it would aid or complement the function of their departments. Interviewees asserted that foragers could partner with the municipality to aid biomass and waste removal (6), and reduce crime by promoting regular, responsible use of open spaces (3). Foraging could also assist biodiversity conservation by promoting the sustainable use of natural resources (3), encouraging the dispersal and planting of indigenous species (3), attracting indigenous animal and bird species (2), and adding human benefits to nature conservation (2). Lastly, it could play a role in encouraging awareness, education, learning, and research about diverse food species (1) and preserving and propagating cultural relationships between plants and people (1).

Even among those who favoured foraging, estimation of sustainable yield and adoption of appropriate harvest practices was a precondition (4), although they felt that fruit foraging was likely to be less damaging than harvesting of other plant parts. There was also the concern that increased food availability could potentially support larger populations of vervet monkeys, which are reportedly a nuisance in Durban (1). There was no significant difference between the stances of interviewees from different departments (χ^2^ = 1.47, p = 0.49).

*‘We would want to try and think about the sustainability of the activity… [And wild edible fruits] to me is a lot more inviting as a space to interact with people… [be]cause… collect[ing] bark for example is clearly a much more destructive activity generally*. *[We ideally want to know] what is it that people want*, *what kind of demand is there for that*, *what is the supply of those resources per protected area*, *and what would be a sustainable offtake*? *And then you could have a monitoring programme to think about whether or not we’ve got the science right*.*’*

- EM2

All interviewees were amenable to the concept of developing products, supply chains, and ecotourism with foraged fruit. The most frequently cited potential benefit of commercial foraging was the creation of employment opportunities, contributing to poverty alleviation, and local economic development (7). Additionally, some proposed that commercial foraging could potentially augment the function of their departments, by realising economic benefits and sustainable resource use in open spaces and conservation areas (5); and by providing favourable indigenous, resilient, and low input alternatives to agriculture, which is a major land use conflict in open space and conservation area management (4). Further, some also noted that commercial foraging could contribute to the culture, knowledge, and uptake of diverse food species (3).

*‘So I think [there is] value in educating the public about weeds which are really nutritional… it [could] be a case of learning about a particular tree and what it can be used for and then encouraging the planting of a particular tree species*. *So we could use this [garden] as a public educational platform to promote the idea*. *Could we have a park in a district… that allows foraging and we actually plant for foraging*?*’*

- PM1

*‘I don’t know if [indigenous fruits] is what people are looking at when they say [agriculture]… but if we think about it in a transformative way*, *because we don’t have to do things like we did in the past*. *We can’t say all we’re going to plant here are apple trees and orange trees*. *We could be planting those various [indigenous fruit] species*.*’*

-EM1

All interviewees concurred that with sustainable practices, commercial foraging would not pose ecological problems. Formalising harvest and sale of foraged fruit through transparent, regulated supply chains would potentially deter unsustainable offtake according to some (3), and could also encourage domestication and cultivation of new species if the demand was high enough (3).

*‘I don’t think [commercial foraging] is risky because that will be [the] same as going to agriculture*. *If there is a demand for those kind of fruits*, *then your nurseries will just come in making sure that the product is cultivated*, *and it’s opportunity*.*’*

- PM3

*‘Now you might have been getting say a tonne [of fruit] a year*, *now you’ve gotta share your tonne*, *and there’s half a tonne each*. *And now somebody else sees it*, *and there’s three or four people*. *Then… is it possible to cultivate these fruits*? *If there’s that kind of demand*, *would it not be easier and better to take it to people*? *[O]nce [wild edible fruits] are all growing around the… homesteads*, *that would be a lot easier*, *with the transport costs as well*. *Now everybody’s got access to their own resource*. *So maybe you take a little bit out the forest*, *naturally*, *but also maybe the more long-term sustainable solution would be to take those trees to people*.*’*

- EM2

Resource allocation in terms of who was eligible to forage in certain areas or from certain trees was recognised as a possible issue (2), but was countered by the observation that wild edible fruit bearing species are fairly common and not ecologically threatened (3). The Durban agroecology division works around resource allocation within citizen groups by encouraging them to create and adhere to a constitution (CS1), and this form of devolved commercial foraging rights may prove useful in urban neighbourhoods or private open spaces.

In the case of intensive foraging in parks or dedicated foraging gardens, the attractiveness of the space may be compromised by foraging (PM1), but design considerations such as designated foraging areas and companion planting could potentially alleviate this issue. Wild foods may be considered as inferior to conventional mass-produced foods, despite their nutritional value, and their relative vitality and productivity in unmanaged landscapes (2).

*‘The Valley Trust… released a poster comparing cabbage to local weeds and things like blackjacks and amaranthus*, *and the nutritional value [of these wild foods] far superseded cabbage*. *So I think that’s the value… in educating the public about weeds which are really nutritional*. *And growing cabbages*, *takes a long time to grow a cabbage… and all the pests* … *And this whole thing of weedy plants being seen as inferior or poor people’s food*. *So the whole connotation that weeds are for poor people*. *Because again that’s where they were trying to promote the value of weeds and foraged foods*.*’*

- PM1

*‘You know weed is in the eye of the beholder… a forager would take this [weed] as fantastic but most people would see those as weeds*.*’*

- EM2

The novelty and seasonality of wild edible fruits were seen as market risks: while they could be a selling point, unfamiliarity and unavailability could also hinder popularity (3). Introducing supply chains to formal markets such as stores and restaurants could help increase exposure and ‘legitimacy’ of wild edible fruits (1), and bridge seasonal gaps in traditional produce (1).

Foraging in urban open spaces is not generally considered illegal unless explicitly stated at the location. Whereas there are some specific concerns, the consensus is that foraging would be beneficial overall to achieving the objectives of all stakeholders.

### 3.7. Environmental worldviews

There was a significant difference between the environmental worldviews of interviewees who were pro-foraging and cautious about encouraging it in public spaces (χ^2^ = 95.77, p<0.001). Notably, those interviewees that were concerned about the implications of urban foraging tended to favour conservation for nature’s sake rather than for human benefit, and tended to believe that pristine nature untouched by humans does exist. Overall, these two statements evoked the most divided responses.

*‘Nature is socially constructed*. *It’s not real*.*’*

- PM1

*‘There are some places where I personally I feel like that the hand of humans should be actually very limited or almost nothing*. *I think that’s really important*.*’*

- EM2

**Fig 3:**
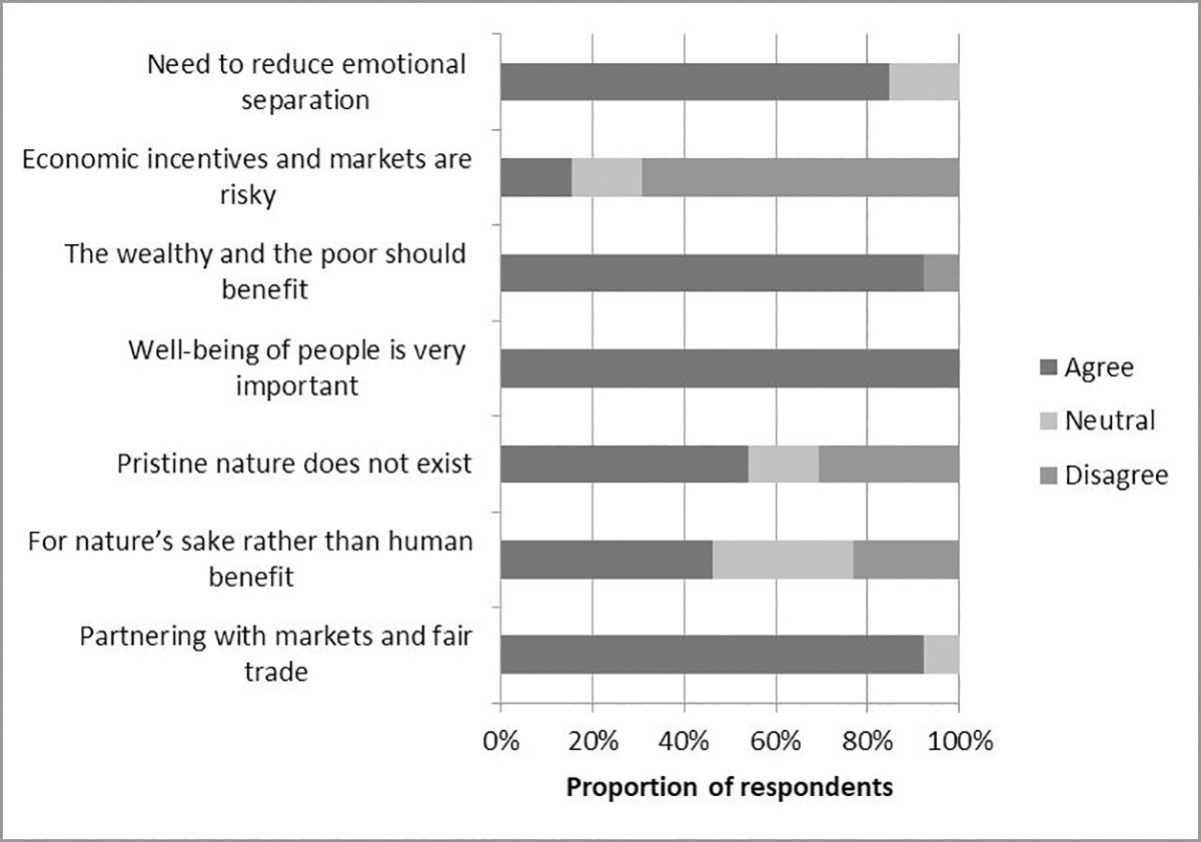
Responses of urban open space managers to the environmental worldview statements Likert scale (n = 13).

All interviewees agreed that the well-being of people is important to nature conservation (Fig 3). Some interviewees expressed the importance of traditional land claims (3) and development rights (3) in urban land use planning in relation to this statement. Twelve of 13 interviewees agreed with partnering with markets and fair trade with a view to conserving nature. Such a partnership was seen as a source of employment opportunities by some (2), and others recognised it as an expression of the value of the ecosystem services that their departments worked towards conserving (4). All but one of the interviewees believed that both the wealthy and the poor should benefit from nature conservation, although it was acknowledged that the rich often benefitted more than the poor (3). Disagreement with this statement (1) echoed the sentiment that even though the rich have larger environmental footprints, they already enjoy more access to nature, and therefore should not hold the same priority as the poor as beneficiaries of nature conservation. While most interviewees believed using economic incentives in nature conservation was not risky, two of them believed it could pose a risk due to uncertainties in the flows and yields of ecosystem services and their sometimes abstract nature.

*‘We have no tangible way of saying this is how this wetland is benefiting X*, *Y*, *and Z person on the ground*. *We can say generally… this is how it may protect us from climate impacts or… provide water*. *But when you put down a development on paper saying I’m gonna employ this many people for this period of time*, *it’s going to result in this much economic development*, *there’s no way we can compete with that*. *As much as it’s important to speak the same language as economists and developers*, *it is difficult*, *a risk*, *because we could come out on the wrong side of that*.*’*

- EM1

*‘We have to ensure that… if we are proclaiming areas for nature conservation*, *areas for obstructing economic use… that they do in fact yield the economic benefits that other forms of the economy do*. *So that’s where I see the risk*. *And you know with nature*, *you aren’t in complete control*. *There’s uncertainties*.*’*

- EM5

Similarly, while most strongly agreed that for nature conservation to better succeed, the emotional separation between people and nature needs to be reduced, two interviewees had a more neutral stance, saying that while emotional connection was important, people’s socio-economic backgrounds and priorities also influenced nature conservation. Some interviewees identified awareness and education as a means of establishing and strengthening this connection (3).

*‘There is the potential*, *if you’re poor or if you don’t have the means*, *yes*, *then surely you could be completely disconnected [from nature] because you can’t get to it*. *But if you’re wealthy enough*, *people seek these opportunities in any case*. *I think [emotional connection to nature] is definitely important*. *I don’t know if it’s completely required*. *I think it definitely helps*. *I mean people will make decisions about pandas or tigers or California rednecks even if you haven’t seen one*.*’*

- EM2

*‘Education plays an important role*. *Some people… don’t know the importance of a tree*, *because nobody told them*, *that it’s not that you mustn’t cut a tree*, *that you get whatever you get from it*. *Sometimes people need to be educated in order to preserve their environment*.*’*

- PM4

## Discussion

The urban municipalities within the IOCB range from small towns to major economic hubs and include the metropolis of Durban. While smaller towns have no formal green spaces, larger municipalities have formal parks and gardens as well as departments dedicated to the maintenance of these. Smaller municipalities manage their informal spaces as a mandate of community services, with budgets and staff in common with waste management and infrastructure development, and often have no specific policy to inform land use or greening decisions (see also [91]). In economic hubs with various land use pressures, municipalities have incorporated open space mapping systems such as the DMOSS [92–94], BOSMAP [82], and the ESMP [80] to prioritise conservation and development. Environment departments in these hubs coordinate collaboration across various stakeholders including developers, town planners, district and provincial departments, NGOs, and citizens with the explicit aim of sustainable land use management, including conservation and restoration. [95–98, pp.122-125, 83, 85, 84, 80].

The limitations of our study include the opportunistic sampling strategy, a posteriori network analysis, and the adaptation of the Future of Conservation framework. Our sample size was limited by the availability and responsiveness of municipal officials, and its representativeness was uneven due to variability in the organisational structure of municipalities. The data for network analysis was gleaned from the interviews as opposed to collected purposively from stakeholders, and weighting was based on the authors’ judgement rather than stakeholder or expert opinion. The abridged version of the Future of Conservation framework was used specifically to compare worldviews on nature conservation and foraging, and does not tie in or compare to the actual framework [55], which is more comprehensive and internally coherent. Our limited and uneven sample size also challenges the validity of the Chi-squared test comparison between the worldviews.

Foraging in urban open spaces covered in the IOCB is legal unless otherwise notified at the site. Some sites require foragers to seek permission from the owners or managers of the space, and this is generally conveyed to the public through signboards. Some public spaces such as the permaculture garden at the Durban Botanic Gardens [99] and Silverglen Nature Reserve [100] are specifically designed with the aim of spreading awareness and propagating resources for sustainable harvesting of indigenous plant resources. In the wider context of urban open space management, foraging is not specifically mentioned or addressed as an activity or an ecosystem service occurring in these spaces, but the broad policy governing these spaces promotes sustainable use of natural resources therein. The policy in coastal KwaZulu Natal is therefore at the very least cognizant of the extractive use of urban open spaces, which is a stark difference from examples in the literature where policy categorically prohibits such use [37, 38]. However, such policy mechanisms exist in only three of the nine municipalities, even though foraging has been observed by interviewees in the nine municipalities. This absence, ambiguity, and in some cases, coarseness of policy has been identified as a challenge by land managers. The lawfulness of foraging may be a concern for foraging and non-foraging citizens with an interest in foraging, and spreading awareness about sustainable foraging may unlock the potential for citizen engagement in land stewardship.

Planting in urban open spaces is undertaken with a clear emphasis on indigenous and useful species. The concept of wild edible fruits is however novel to most land managers, and less than a handful of these species are currently included in plantings. In Durban, wild edible fruits may prove particularly useful to the environment department, which seeks use-based conservation strategies in protected and traditional areas, and for the agroecology division that seeks low-input resilient food species. Thus, planting indigenous wild edible species for foraging could provide a win-win solution for biodiversity conservation and food production, which may conventionally be perceived as being conflicting land uses [101, 102]. Urban parks are often host to high species richness, although a considerable portion of their species may be exotic [103, 104]. The medium-sized and small municipalities could benefit from incorporating indigenous edible species in planning their upcoming green infrastructure to be multifunctional [105]. Providing households with indigenous fruit tree species also has the potential to perpetuate traditional knowledge and prevent the extinction of experience of biocultural diversity [34].

Decisions on planting are influenced by various stakeholders in different contexts. In Durban, the agroecology, environment, and parks departments are the main providers of plants and planting-related funding. In the medium sized municipalities, these are supplied by the environment department, the DAFF, and the EDTEA, and in the small municipalities, they are supplied by NGOs, the community services department, and the EDTEA. Informing these providers about the potential of wild edible fruits and the target areas for their planting would improve outcomes for all stakeholders. In the metropolitan and medium-sized municipalities, the environment and parks departments are the main mobilisers in open space management, whereas in small municipalities, the community services department and NGOs play a more central role (Section 3.4). These stakeholders will play a crucial role in disseminating awareness about sustainable foraging and the additional roles foragers could play in assisting the local administration.

All municipalities face challenges with waste management and illegal activities in their open spaces, as well as shortage of staff and budgets to manage these spaces, a finding echoed by Gwedla and Shackleton [91]. Foragers could fill this gap directly by being voluntary monitors and reporters of these issues. Existing citizen collaborations could be bolstered by foragers, who are regular and active users of open spaces, hold diverse knowledge about, and values for, these spaces, but may not have a legitimate voice or representation in local governance [21, 31,33]. Land managers are optimistic about exploring the potential of foragers as active partners in land use management. They identified a number of ways in which foraging fits favourably into their land use frameworks and the wider goals of the local and national administration. With appropriate consideration to local context and stakeholder objectives [35, 36], municipalities can form mosaic governance partnerships to better achieve specific goals [33].

Recognising the social and economic value of foraging grounds could build a case for improved collaborative stewardship of existing informal green spaces, and for enrichment of existing green and degraded spaces with useful species. Clear and detailed evidence of the economic contribution (e.g.[16,24]) as well as cultural connotations (e.g. [11, 13, 22, 31]) is required to help ascertain these values, and also to identify priority areas (e.g. [28, 30]) for planting, conservation, and restoration. Quantification of some of these values and justification of land use decisions based on them can be difficult especially when competing with economic development (Section 3.7). Formalising and commercialising certain foraged products may be a way of representing tangible yields from green spaces, although this may in some cases conflict with the anti-capitalistic ideology of certain foragers [20–22]. More information on foragers’ perspectives and motives is required in order to develop partnerships and co-operative enterprises as applicable.

Urban land managers are amenable to the concept and activity of foraging contingent upon adherence to use of common species and sustainable harvest practices and quantities based on scientific evidence. Wild edible fruit foraging is perceived as a more viable alternative to other forms of potentially more damaging extraction (e.g. debarking, lopping). This is empirically true [61, 106], but different species respond differently to varying degrees of harvest [62], and therefore all stakeholders should be equipped with knowledge on best practices including low-impact harvest and the quantities that may be safely harvested for each species. In some cases, locals who have used the species over generations may already possess appropriate traditional knowledge about sustainable harvest [107]. Further considerations related to foraging include designing foraging spaces and tenure systems to minimise overexploitation and user conflicts, and leveraging the unfamiliar and variable nature of wild foraged fruits to increase uptake by casual users as well as market buyers.

The environmental worldviews results indicate that land managers’ normative perceptions of pristine nature and its conservation may influence their decision to plant and advocate for foraging (Section 3.7). The construct of pristine nature and its conservation by excluding humans is a major polarising point among conservation workers worldwide [55]. However, although land managers had different positions on the concept of pristine nature and conserving its intrinsic value, they all conceded that with appropriate measures, casual, cultural, and commercial foraging could be sustainable. This is in consonance with findings from a global survey in which conservation workers in Africa favoured people-centric and capital-based conservation [55].

## Conclusions

Foraging is a permissible activity in most urban open spaces in the study area, with tacit support from policy and land managers. Lack of information about wild indigenous edible species and their sustainable harvest is the main barrier to planting of foraging-friendly species. Planting for foraging has the potential to provide multiple benefits for all stakeholders, including fulfilling biodiversity conservation and food security objectives. Based on our findings, we suggest that exchange of information between stakeholders on the nature of foraging spaces, species and allied activities could improve co-management of urban green spaces. A better understanding of the motives behind foraging and the values associated with the urban green spaces it is undertaken in will aid the development of governance partnerships and potential incentives for sustainable use and conservation of urban green spaces.

## Supporting information

S1 Appendix (DOCX)

S1 Dataset (XLSX)
