# Supplementary material for: Urban foraging: Land management policy, perspectives, and potential
Source: PLoS One. 2020 Apr 7;15(4):e0230693. doi: 10.1371/journal.pone.0230693 (PMC7138303; doi:10.1371/journal.pone.0230693)
Supplement: S1 Appendix — (DOCX) [file pone.0230693.s001.docx]

## Appendix 1

1. Open spaces
   1. What is the area covered by open spaces in the municipality? What types of open spaces does the municipality have (informal, formal, developed/improved, specialised, conservation areas)? By whom are these open spaces managed?
   2. What are the management practices undertaken in these open spaces? Are there policies addressing the nature of public use of these spaces?
   3. What are the challenges faced by the municipality in managing these spaces? Are there proactive efforts towards engaging the public in overcoming these challenges?
2. Planting
   1. What is the average frequency and volume of planting within the municipality? What are the target areas for such planting? What are the criteria for selection of species to plant?
3. Foraging
   1. Does foraging occur in the municipality? Have the officials observed such activity? Do they believe it occurs? Who forages where and for what?
   2. Would the municipality encourage foraging in open spaces? Would they encourage commercial scale foraging? Why?
4. Environmental Worldviews: To what extent do you agree with the following statements?

| Statement | Agree | Neutral | Disagree |
| --- | --- | --- | --- |
| Nature can be conserved by partnering with markets and fair trade. |  |  |  |
| Nature should be protected for nature’s sake rather than human benefit. |  |  |  |
| Pristine nature untouched by human influence does not exist. |  |  |  |
| The well-being of people is very important in conserving nature. |  |  |  |
| Both the wealthy and the poor should benefit from nature conservation. |  |  |  |
| Using economic incentives and markets in nature conservation is risky. |  |  |  |
| We need to reduce the emotional separation between people and nature. |  |  |  |
